# Ethnomedicinal plants used for the prevention and treatment of anemia in the Philippines: a systematic review

**DOI:** 10.1186/s41182-023-00515-x

**Published:** 2023-05-12

**Authors:** Mariel C. Magtalas, Patrick Tracy Balbin, Elljhay C. Cruz, Richard F. Clemente, Ara Karizza G. Buan, Jervy P. Garcia, Ka Yiu Lee, Ourlad Alzeus G. Tantengco

**Affiliations:** 1 Biology Department, College of Science, Bulacan State University, Malolos, Bulacan Philippines; 2 College of Medicine, University of the Philippines Manila, Manila, Philippines; 3 Department of Family and Community Medicine, Eastern Visayas Medical Center, Tacloban City, Philippines; 4 Department of Health Sciences, Mid Sweden University, Östersund, Sweden; 5 Department of Physiology, College of Medicine, University of the Philippines Manila, Manila, Philippines; 6 Department of Biology, College of Science, De La Salle University, Manila, Philippines

**Keywords:** Ethnobotany, Anemia treatment, *Ipomoea*, Toxicologic, Teratogenic, Decoction

## Abstract

**Background:**

Medicinal plants are still used in developing countries, including the Philippines, to treat common diseases in the community. Anemia is a common disease encountered in the community. It is characterized by a lower-than-normal level of red blood cell count. This systematic review identified the medicinal plants used for anemia treatment in the Philippines.

**Methods:**

The study was conducted based on the PRISMA flow diagram, starting with a data search on electronic databases. The collected studies were screened based on the inclusion and exclusion criteria. The necessary information was extracted from the eligible research papers, and the studies’ quality was assessed through a developed quality assessment tool.

**Results:**

A total of 20 ethnobotanical studies on medicinal plants used for anemia treatment were obtained from different provinces within the 12 regions of the Philippines. Most ethnobotanical studies were conducted in Region X (Northern Mindanao), CAR (Cordillera Administrative Region), and Region XIII (CARAGA), Philippines. The most common plant family is Convovulaceae, with nine records (21.95%), followed by Cucurbitaceae, with six records (14.63%), and Moringaceae, with five records (12.2%). The most common plant part used was the leaves. Others involved mixing  different plant parts, with fruits and leaves being the most common combination. The most common route of administration utilized was drinking the decoction, followed by eating the plant. Most medicinal plants used to treat anemia in the Philippines had records of toxicologic (four species, 15.38%) or teratogenic (one species, 3.85%) properties. Eight plant species were reported as nontoxic (30.77%). In addition, ten plant species (38.46%) had no data on toxicity or teratogenicity.

**Conclusion:**

There were only 20 ethnobotanical studies that documented the use of plants in treating anemia in the Philippines. This study listed several medicinal plants used in treating anemia in the Philippines. However, pharmacological and toxicological studies are still needed to determine their safety and efficacy in treating anemia in the community.

**Supplementary Information:**

The online version contains supplementary material available at 10.1186/s41182-023-00515-x.

## Introduction

The Philippines is one of the mega-diverse countries in the world, having 75% of the world’s biodiversity, both in flora and fauna [1]. With rich flora, the Filipinos explored the use of these plants to treat diseases. This indigenous practice has been part of Filipino indigenous groups’ culture and has been handed down for generations [2]. Traditional medicine has remained popular for centuries, with around 80% of indigenous people still dependent on conventional medicine [3]. With the continuing reliance on more readily accessible plant-based medicine, rural dwellers sought out “parahilots”, town folk osteopathy practitioners [4], to cure diseases [2]. Several ethnobotanical studies surveyed the preparation and use of medicinal plants by geographically isolated tribes in the country [3]. However, most ethnobotanical studies in the Philippines only listed medicinal plants and their use. There were not a lot of ethnopharmacological investigations to validate the results of ethnobotanical surveys [5].

One of the prevailing conditions in the Philippines for which medicinal plants are still used is anemia. Anemia is characterized by a lower-than-normal red blood cell count level that leads to decreased oxygen blood levels, hemoglobin function abnormalities, and reduced hemoglobin and/or hematocrit content [6]. Based on the recommended cutoff set by the World Health Organization, anemia still affects one-third of the world’s population, with significant variations in low- and middle-income countries. It has now been considered a primary public health concern worldwide, particularly affecting certain vulnerable groups in developing countries with a high prevalence of poverty, malnutrition, and parasitism [7]. In 2019, it was estimated that there were 1.8 billion cases of anemia worldwide, which was more prevalent among women in all age groups [8].

The top three causes of anemia are beta-thalassemia, iron deficiency, and vitamin A deficiency. Most cases in the Philippines are caused by iron deficiency anemia (IDA). Identified causes are the lack of iron-rich foods and iron-fortified products [9]. In a local survey conducted in March 2021, the highest prevalence of IDA was recorded in pregnant women, followed by the geriatric (> 60 years) and children less than 5 years of age [10]. The consequences of anemia include high morbidity and mortality rates, particularly in women and children [11, 12], increased incidence of poor birth outcomes such as low birth weight and maternal and perinatal mortality [13, 14], poor physical performance in adults [15], cognitive and behavioral impairment in children [16], and compromised immune status [9].

Treatment for anemia largely depends on the identified cause, severity, patient characteristics, individual preferences, and geographical and cultural acceptance. Understanding and establishing anemia’s complex and diverse etiology is crucial to providing appropriate interventions. These interventions may not be limited to synthetic or commercially available drugs but should also cover medicinal plants used locally to treat anemia. Research on the use of medicinal plants in treating anemia in the Philippines is still limited, with the available data restricted only to small-scale studies among community dwellers in various parts of the country. In a survey conducted by Susaya-Garcia et al. (2018) [17], *Momordica charantia* L. and *Moringa oleifera* Lam. have been used by residents of remote villages in a small town in Jaro, Leyte, to treat anemia [17]. Similar studies have likewise found *Alternanthera sessilis* (L.) DC. [18], *Lagerstroemia speciosa* (L.) Pers., and Convolvulaceae *Ipomoea sp.* as anemia treatments among Ayta communities in Dinalupihan, Bataan [19]. In a survey of medicinal plants used by traditional healers in Pagadian City, *Ipomea batatas* (L.) Lam and *Caesalpinia sappan* L. are used in treating anemia [20]. Meanwhile, the indigenous Panay Bukidnon group utilizes *Hibiscus acetosella* Welw. ex Ficalho for anemia [21]. These studies documented the use of several medicinal plants in the country. However, only ten medicinal plants were clinically validated by the Philippine Institute of Traditional and Alternative Health Care (PITAHC) under the Department of Health (DOH) of the Philippines, and none were approved for anemia. Hence, this systematic review aims to identify the medicinal plants used to treat anemia in the Philippines as a reference list for plants that have yet to be clinically validated or recognized. Additionally, this review also included toxicologic and teratogenic data on plant species that can be used as a reference for their safety and the need for further testing.

## Methods

### Study selection

The PRISMA flow diagram [22] was used to guide this systematic review. From inception to December 5, 2021, a systematic literature review was conducted in three electronic databases: Ovid Medline, Scopus, and EBSCO CINAHL. A manual search was also conducted on Google Scholar. This search strategy was used in Scopus, EBSCO CINAHL, and Google Scholar: (ethnobot* OR ethnomed* OR ethnopharmacolo* OR “medicinal plan*”) AND (Philippin* OR Filipin*). For Ovid Medline, this search strategy was used: (ethnobotany OR ethnomedicine OR ethnopharmacology OR medicinal plants) AND (Philippines OR Filipino). However, this protocol was not registered in PROSPERO or other databases for systematic review and meta-analysis. The articles included in the study were only those written in English or Filipino. Observational studies were of particular interest because they provided primary information about ethnobotanical knowledge. Systematic reviews, literature reviews, letters to the editor, comments, and case reports were excluded.

### Data extraction

Four reviewers **—**MCM, PTB, ECC, and RCG**—** independently reviewed the titles and abstracts. For each eligible study, full-text articles were obtained. Afterward, full-text articles were independently evaluated. All irrelevant articles were removed, and the reasons for their removal were documented. The following information was gathered from each included study: first author; year of publication; plant species; plant part used; method of preparation; route of administration; traditional use; ethnic group or users; and place of study. The results of this study were presented as tables and graphs generated using Microsoft Excel.

### Assessment of the study quality

We used a previously developed quality assessment tool for ethnobotanical studies by Magtalas et al. (2022) [23]. This tool was adapted from a study by Timmer et al. (2003) [24]. It was tailored for ethnobotanical research and assessed the quality of ethnobotanical research as low, acceptable, or high (Table 1). The quality assessment tool was composed of ten questions evaluating the quality of the study objectives, the study design, the completeness of the description of the study area and population, the details of the methods that will allow replication of the study, the calculation of the sample size, the taxonomic classification of plants used in the study, a sufficient explanation of the results, and whether the results support the conclusions. However, it is vital to note that an assessment of the risk of bias in each study was not performed.

**Table 1:** The quality assessment tool used for determining the quality of reports included in this systematic review

Quality Assessment Tool	
**Scoring:** Fully compliant = 2 points Partially compliant = 1 point Not compliant = 0 N/A = not applicable	**Total score:** 17–20 = High quality 11–16 = Regular quality 0–10 = Low quality
1. Are the questions or objectives sufficiently described?	
2. Is the study design appropriate to answer the study question/s?	
3. Is the study area and population sufficiently described?	
4. Are the methods described in sufficient detail?	
5. Can the study be easily replicated?	
6. Is the sample size of informants sufficient or justified?	
7. Are the medicinal plants verified by a taxonomist?	
8. Did the paper provide appropriate descriptive and quantitative analysis?	
9. Are the results reported in sufficient detail?	
10. Do the results support the conclusion?	

## Results

**Fig. 1:**
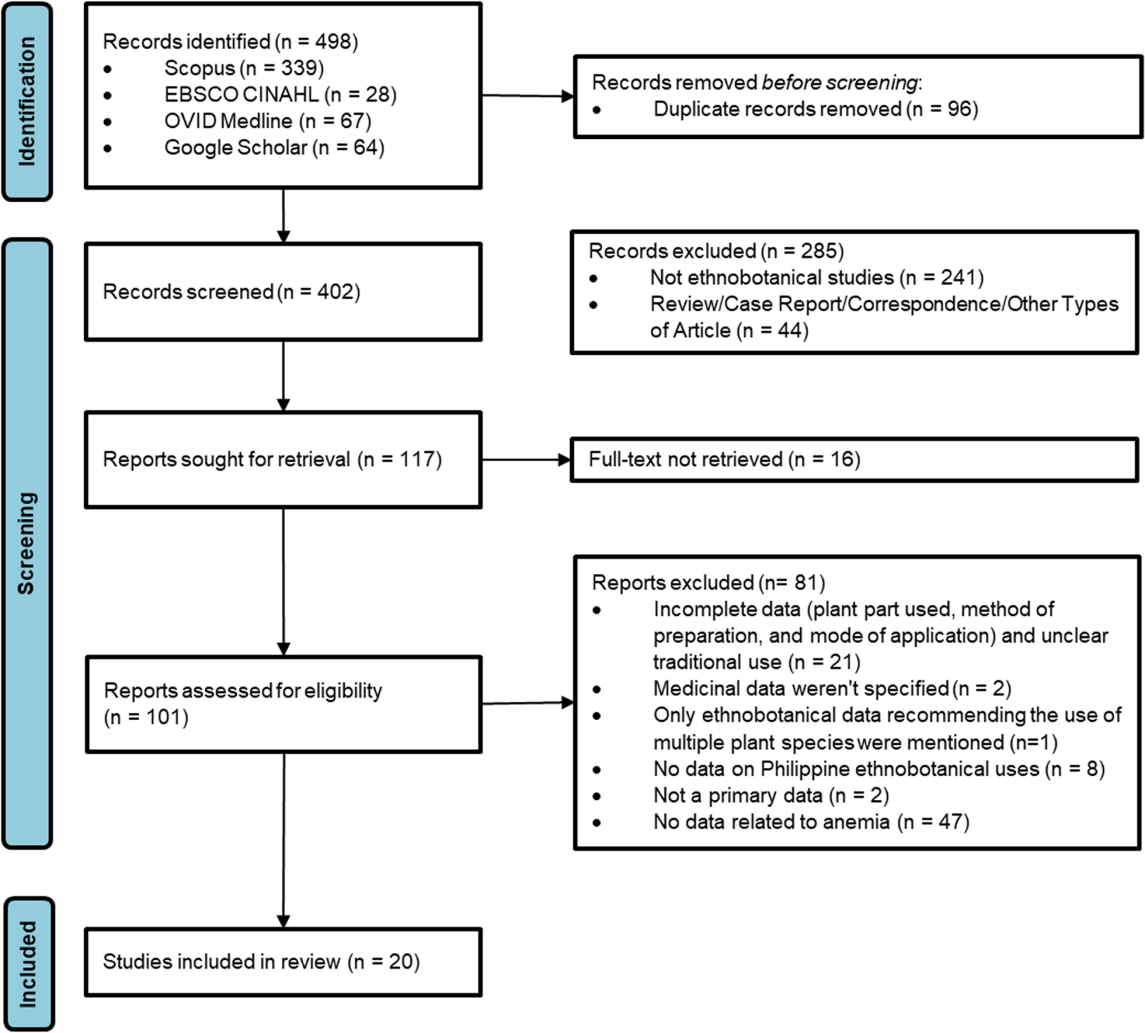
PRISMA flowchart of the study. Eligible studies were included based on the following inclusion criteria: (i) ethnobotanical studies conducted in the Philippines; (ii) the study included medicinal plants used in the management of anemia; (iii) the study has sufficient data regarding the species of the medicinal plants, parts used, method of preparation, and route of administration

The initial data search provided 498 studies, with 434 obtained from Scopus (68.07%), 28 from EBSCO CINAHL (5.62%), and 67 from OVID Medline databases (13.45%). In addition, 64 studies (12.85%) were obtained from the manual search on other platforms, including Google Scholar. After full-text analysis, only 20 studies were included in the qualitative synthesis (Fig. 1). The qualitative synthesis and summary of the full-text analysis of the studies included in this review are shown in Table 2 and Additional file 1: Table S1, respectively.

**Table 2:** A qualitative synthesis of the studies with data on plants used for anemia in the Philippines

First author and year	Study design	Province	Informants	Sample size	Number of plant species used in anemia
Abe (2013) [36]	Observational Study	Batanes	Ivatan	116	6
Alduhisa (2019) [39]	Observational Study	Misamis Occidental	Subanen	83	1
Balangcod (2011) [40]	Observational Study	Ifugao	Kalanguya tribe	150	1
Balangcod (2015) [37]	Observational Study	Benguet	Ibalois	80	2
Balangcod (2018) [41]	Observational Study	Benguet	Locals	107	2
Bodner (1988) [25]	Observational Study	Mt. Province	Bontoc tribe	Not stated	1
Caunca (2021) [42]	Observational Study	Cavite	Local herbalists	94	1
Dapar (2020) [43]	Observational Study	Agusan del Sur	Manobos	335	4
de Guzman (2020) [44]	Observational Study	Zamboanga Sibugay	Local herbalists	30	1
Ducusin (2017) [45]	Observational Study	La Union	Indigenous people	40	4
Flores (2016) [46]	Observational Study	Quiapo	Buyers and sellers	39	1
Langenberger (2009) [47]	Observational Study	Leyte	Male farmers	6	2
Naive (2021) [48]	Observational Study	Bukidnon	Talaandig tribe	19	5
Odchimar (2017) [49]	Observational Study	Bukidnon	Talaandig tribe	Not stated	1
Olowa (2012) [50]	Observational Study	Lanao del Norte	Higaonons	65	1
Olowa (2015) [51]	Observational Study	Lanao del Norte	Maranaos	228	3
Ong (2014) [52]	Observational Study	Guimaras	Ati Negritos	65	2
Paraguison (2021) [53]	Observational Study	Agusan del Sur	Manobos	144	1
Rubio (2018) [54]	Observational Study	North Cotabato	Traditional practitioners	20	1
Tantengco (2018) [19]	Observational Study	Bataan	Aytas	26	1

### Annual trends in the number of ethnobotanical studies

**Fig. 2:**
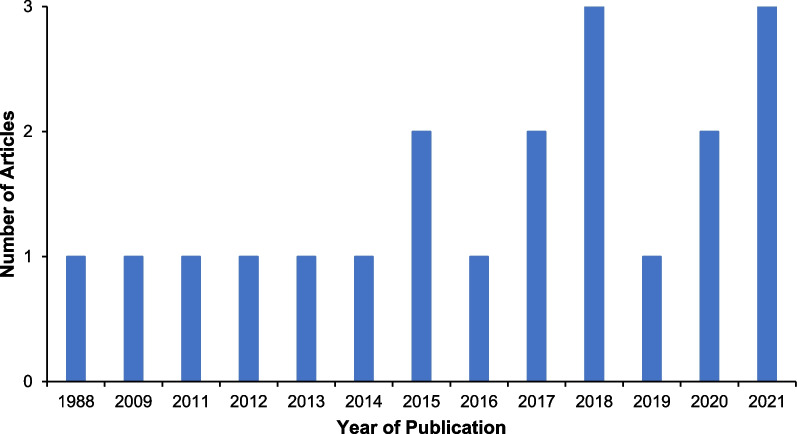
Annual trend of ethnobotanical studies with data on plants that prevent and treat anemia in the Philippines

Ethnobotanical studies on anemia treatment in the Philippines gradually increased in the past years. The oldest data recorded was a study by Connie Cox Bodner and Roy E. Gereau [25], entitled “A contribution to Bontoc ethnobotany”, which pioneered the studies in this field. Between 1988 and 2014, research activities were still limited, with only six related studies documented. However, beginning in 2015, there was an alternate rise and fall in the published literature before it peaked in 2018 with three research studies. In contrast, there was a significant drop in 2019, which quickly recovered a year later, with three publications. Though the trend suggested a continuously growing number of studies performed on ethnobotanical medicine throughout the years, there is still a significant gap between the number of published research and the number of ethnic groups in the Philippines that must be studied (Fig. 2).

### Geographical distribution of studies

**Fig. 3:**
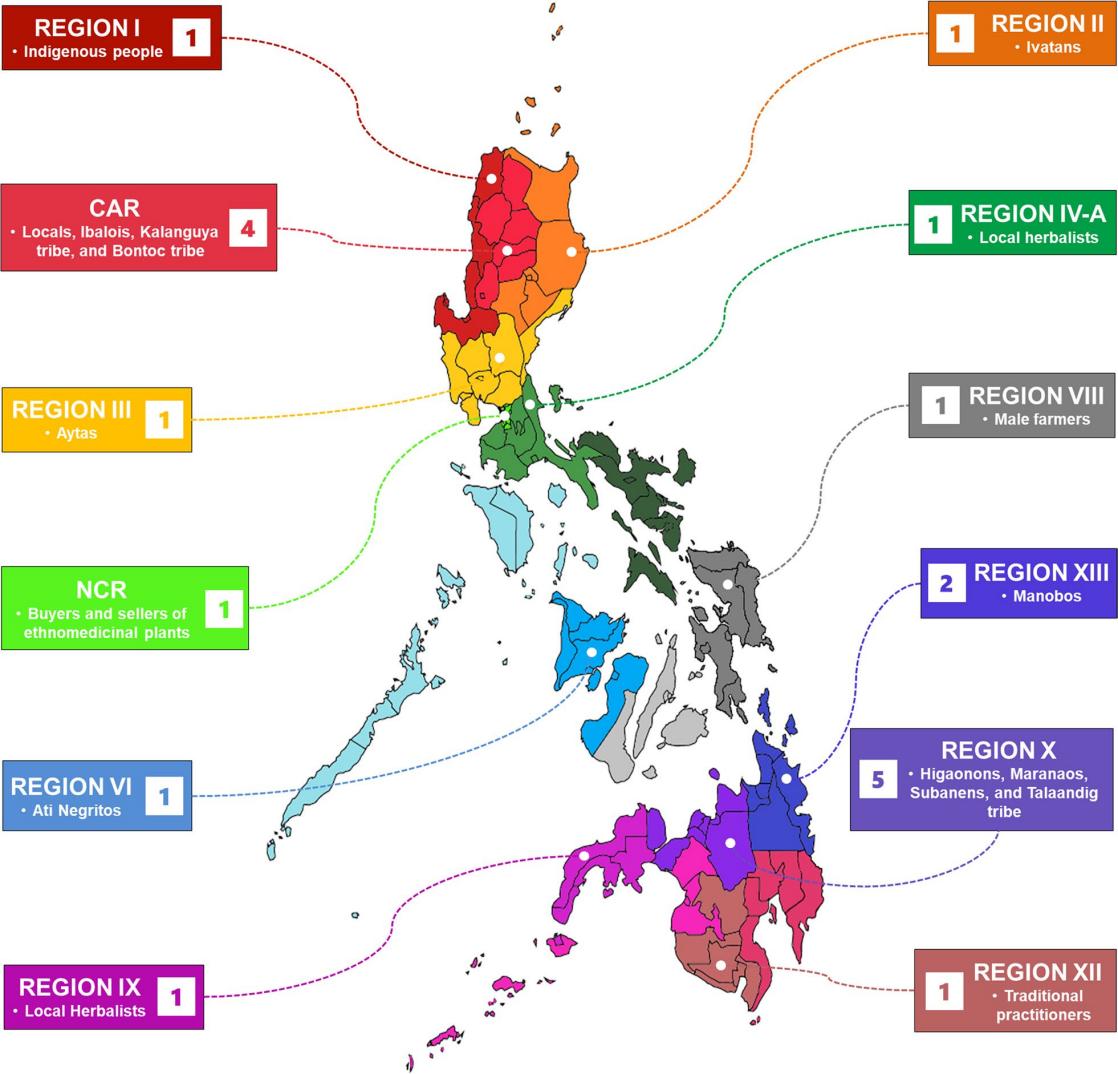
Geographical distribution of the studies with data on plants used to prevent and treat anemia per region in the Philippines and the ethnic groups/users stated in the studies per region

The ethnobotanical studies on medicinal plants used for anemia treatment were obtained from different provinces within the 12 regions of the Philippines. Region X (Northern Mindanao), home to the Higaonons, Maranaos, Subanens, and Talaandig tribes, had the highest number of studies, with a total of five (25%) papers originating from the region. Region X was followed by the Cordillera Administrative Region (CAR) with four (20%), Region XIII (Caraga) with two (10%), and with only one (5%) ethnobotanical study, each originating from Region I (Ilocos Region), Region II (Cagayan Valley), Region III (Central Luzon), Region IV-A (CALABARZON), the National Capital Region (NCR), Region VI (Western Visayas), Region VIII (Eastern Visayas), Region IX (Zamboanga Peninsula), and Region XII (SOCCKSARGEN). Meanwhile, no research outputs were obtained from Regions IV-B (MIMAROPA), V (Bicol Region), VII (Central Visayas), XI (Davao Region), and the Bangsamoro Autonomous Region in Muslim Mindanao (BARMM) (Fig. 3).

### Quality assessment of the studies

**Fig. 4:**
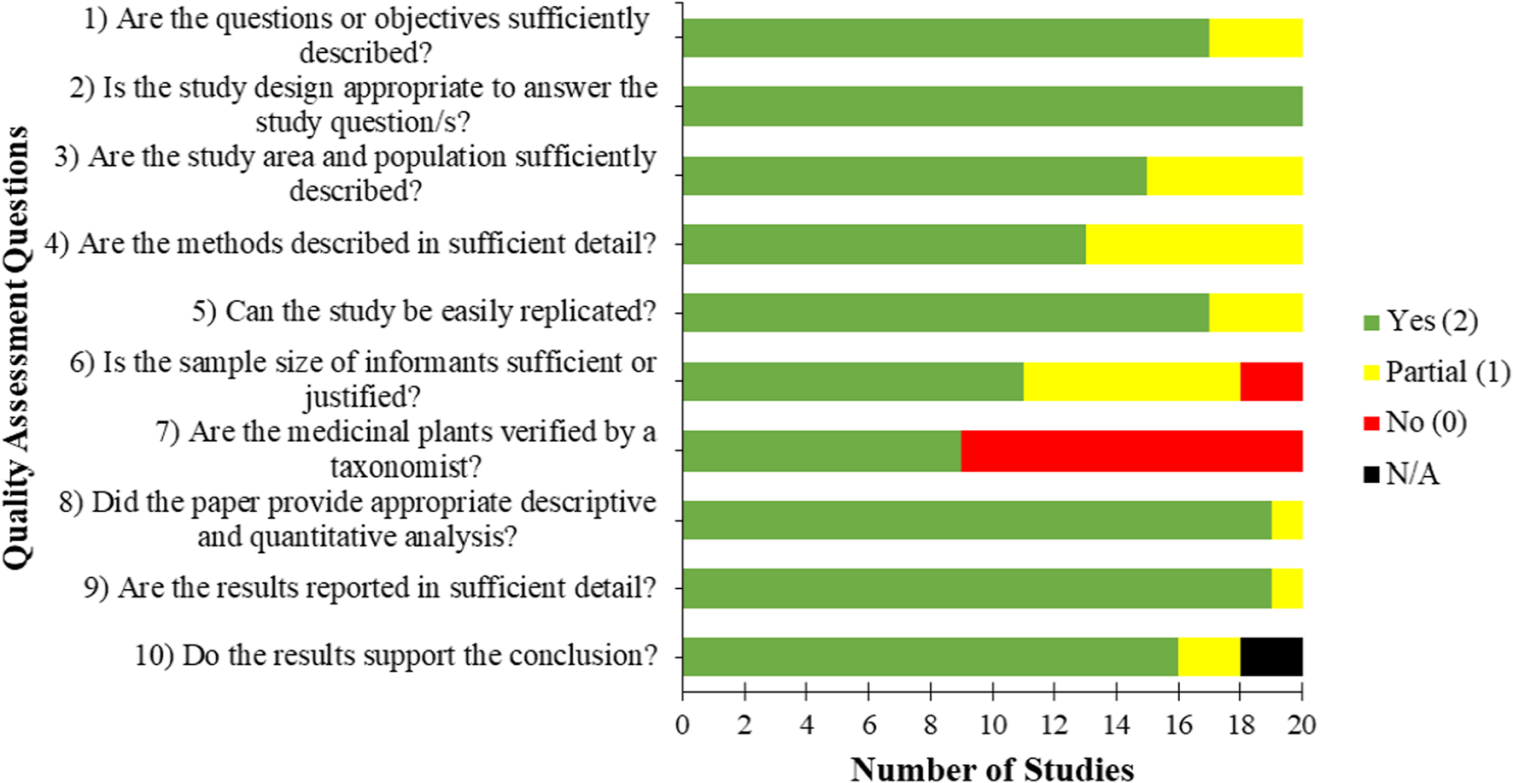
Quality assessment of the studies with data on plants used to prevent and treat anemia in the Philippines. Scoring: fully compliant = 2 points; partially compliant = 1 point; No = 0; N/A = not applicable. Total score: 17–20 = high quality; 11–16 = acceptable quality; 0–10 = low quality

The result showed that out of the 20 ethnobotanical studies obtained, 17 (85.0%) of the papers reviewed were of high quality. Meanwhile, the remaining three (15.0%) papers had regular or acceptable quality, implying that all papers gathered have good quality and reproducibility. Unfortunately, nine (45.0%) papers did not have their plant specimen verified by a taxonomist (Fig. 4; Additional file 1: Table S2).

### Most common plant families, genera, and species of medicinal plants used to treat anemia in the Philippines

**Fig. 5:**
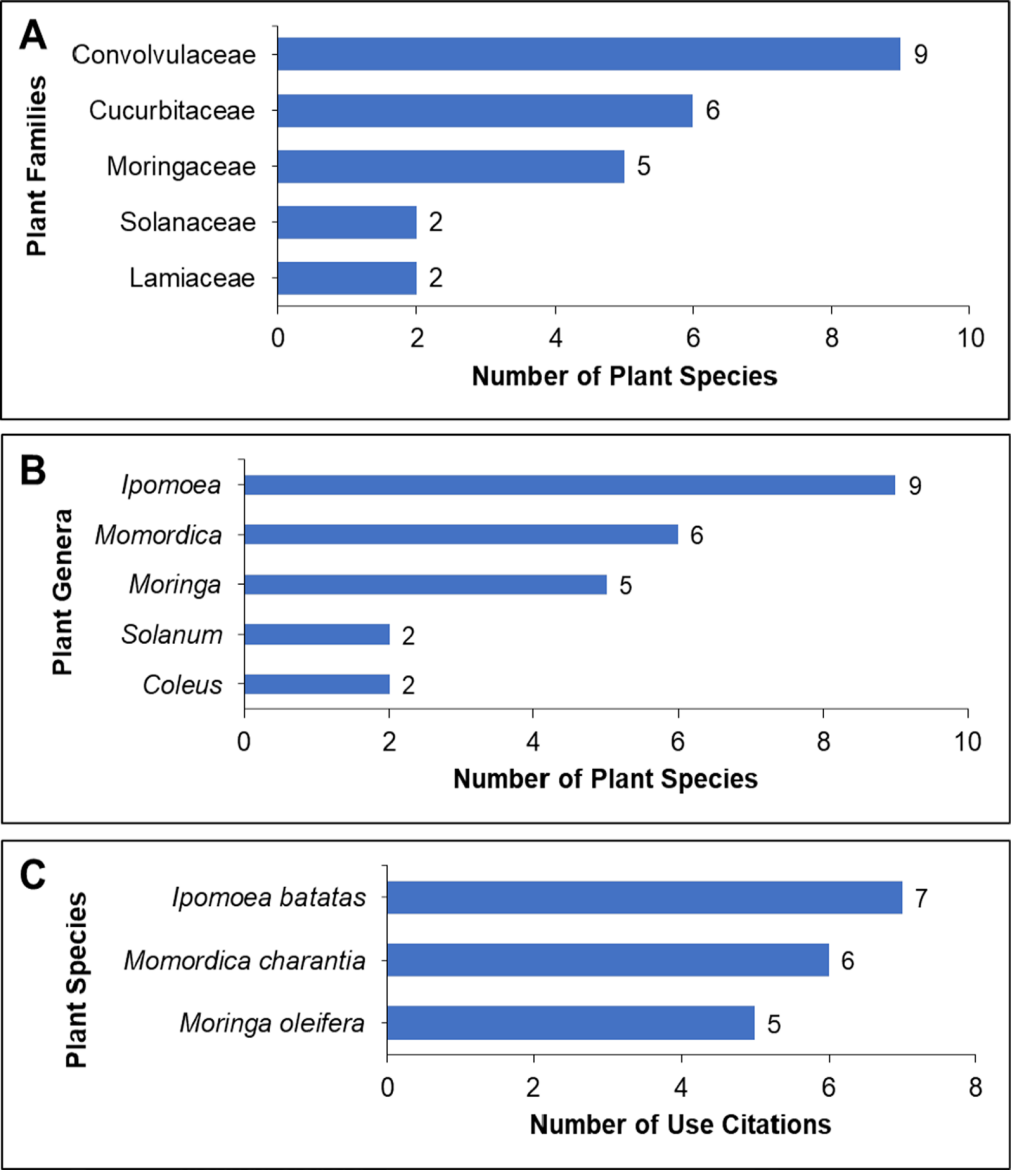
Most common families (**A**), genera (**B**), and species (**C**) of plants used for the prevention and treatment of anemia in the Philippines

A total of 22 plant families, 22 genera, and 26 species were included in this systematic review. Convovulaceae was the most frequently mentioned plant family, with nine records (21.95%), followed by Cucurbitaceae with six records (14.63%), Moringaceae with five records (12.2%), and Solanaceae and Lamiaceae with two records each (4.88%). (Fig. 5A). The most common plant genera (Fig. 5B) were *Ipomoea*, with nine records (21.95%), *Momordica*, with six records (14.63%), *Moringa*, with five mentions (12.2%), and *Solanum* and *Coleus*, with two records (4.88%) each. Similarly, *I. batatas* (L.) Lam. was the most frequently mentioned plant species, with seven records (17.07%), followed by *Momordica charantia* L., with six (14.63%), and *Moringa oleifera* Lam., with five (12.2%) records (Fig. 5C).

### Most common plant parts used, route of administration, method of preparation of medicinal plants used to treat anemia in the Philippines

**Fig. 6:**
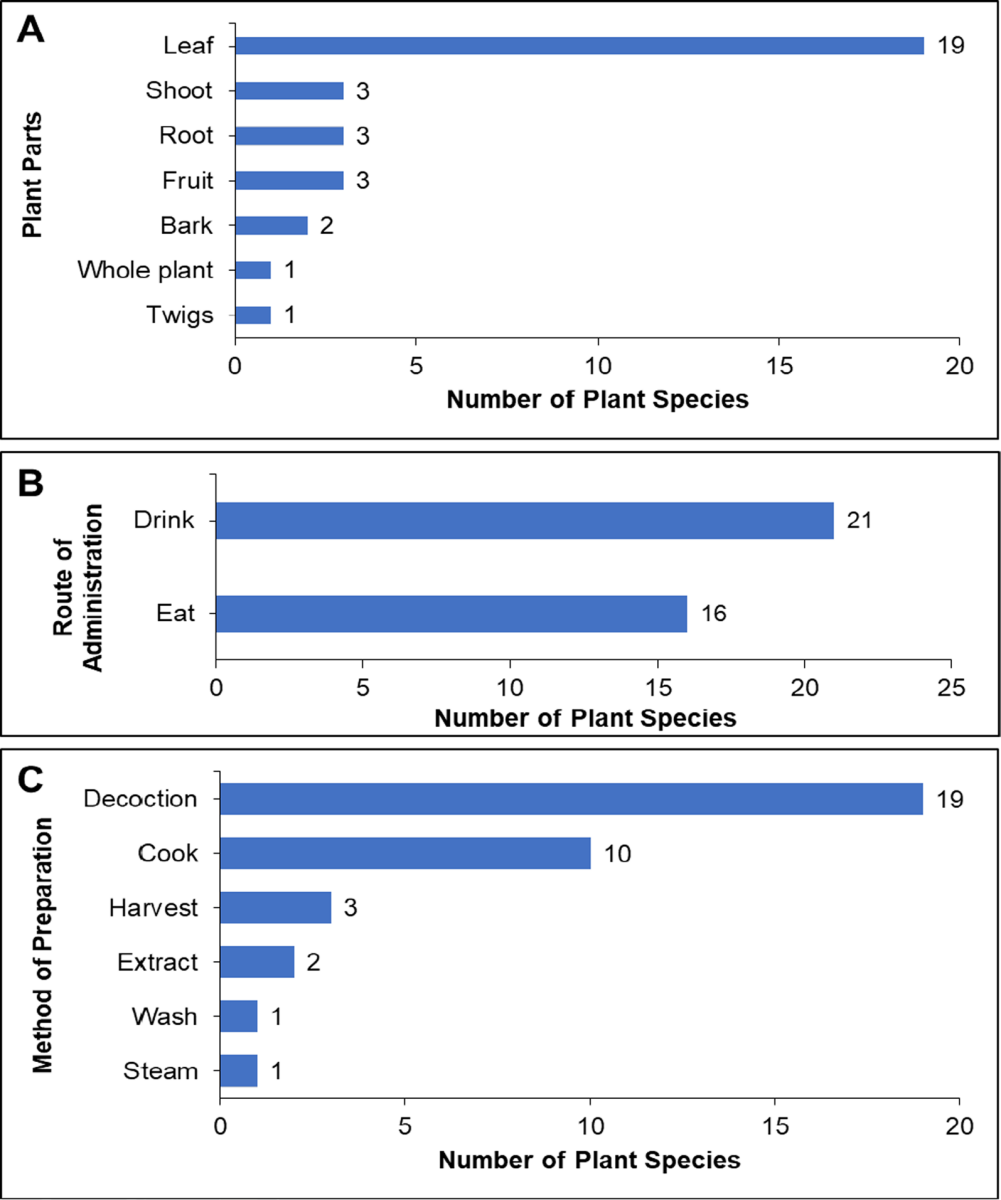
Most common plant parts (single) (**A**), routes of administration (**B**), and methods of preparation (**C**) of plants used for the prevention and treatment of anemia in the Philippines

The whole plant, single plant parts, or mixed plant parts can be used in medicinal plant preparations to treat anemia. Thirty-two (75.61%) ethnobotanical records used only a single plant part, and one used the whole plant itself (2.44%). The most common single plant part used was the leaf (19 records, 46.34%), followed by the shoots, roots, and fruits (three records each, 7.32%), then the bark (two records, 4.89%), and lastly, the twig (one record, 2.44%) (Fig. 6A). Meanwhile, nine records involved the use of mixed plant parts, with fruit and leaf as the most common combination (three records, 7.32%), followed by leaf and shoot (two records, 4.89%), leaf and seed (two records, 4.89%), and the less common bark and leaf (one record, 2.44%) and bark, flower, and seed (one record, 2.44%) combinations (Additional file 1: Figure S1A).

The route of administration was grouped into two categories: (1) plants with a single route of administration (32 records, 90.24%) (Fig. 6B), and (2) plants with two or more steps followed for the administration (three records, 7.32%) (Additional file 1: Figure S1B). The single routes of administration utilized were as follows: drinking (21 records, 51.22%), followed by eating (16 records, 39.02%). Meanwhile, for the second category, one record (2.44%) for each route of administration was recorded. These two-step administrations included: (1) eat cooked leaves as a vegetable; drink a decoction of leaves; (2) eat cooked leaves and drink juice; (3) eat cooked leaves or fruits as a vegetable; drink a decoction of leaves; and (4) eat fresh or cooked leaves or seeds as vegetables; drink a decoction of leaves.

The methods of plant preparation were divided into two categories: (1) plants prepared in a single step with 36 records obtained (Fig. 6C); and (2) plants prepared in two separate steps to produce two separate products with five records obtained (Additional file 1: Figure S1C). For the plants prepared in a single step, the most common method employed is decoction (19 records, 46.34%), followed by cooking (ten records, 24.39%), then harvesting (three records, 7.32%), extracting (two records, 4.88%), then washing (one record, 2.44%), and steaming (one record, 2.44%). One record was obtained for each of the multi-step methods of preparation, which include: (1) cooking leaves and fruit to make a decoction of the leaf; (2) cooking leaves and seed to make a decoction of leaves; (3) cooking and decoction; (4) cooking or juice (squeezing to get sap); and (5) decoction, juice, and cooking for the second category.

### Toxicity and teratogenic properties of medicinal plants used to treat anemia in the Philippines

**Fig. 7:**
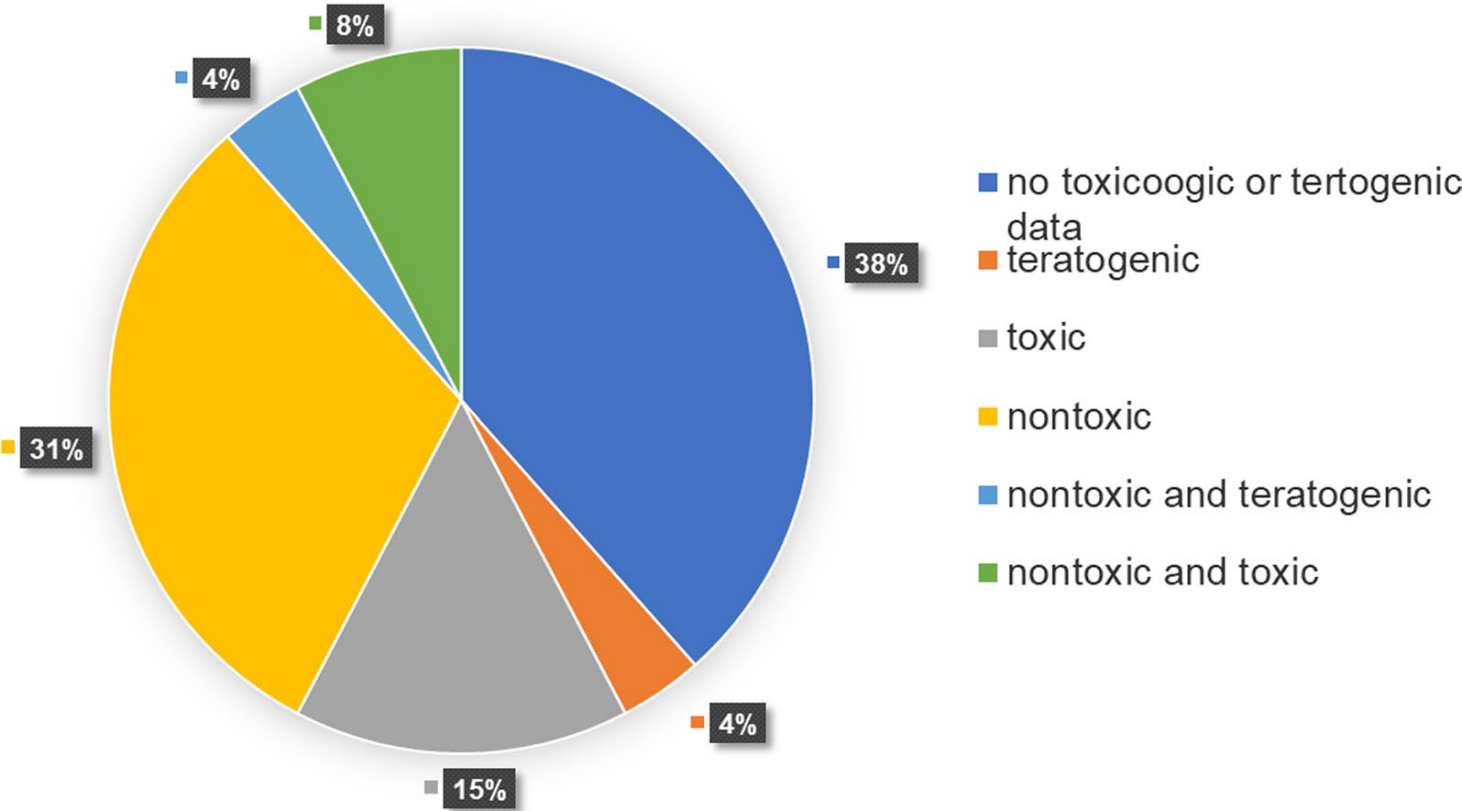
Toxicologic and teratogenic data of plants used to prevent and treat anemia in the Philippines

The toxicologic and teratogenic data of the plants used for anemia were determined through a literature search to provide information on the safety of these medicinal plants. Sixteen (61.5%) plant species had toxicologic and teratogenic data, whereas ten plant species (38.5%) did not (Additional file 1: Table S3). Various methods were utilized to assess the toxicity and teratogenicity of plant species used to treat anemia. Of the 19 reviewed preclinical studies for the toxicity and teratogenicity of the plants, 15 (78.94%) were conducted in vivo, and four (21.05%) were in vitro. The most common animal model used was Wistar rats (seven records, 36.8%), followed by Swiss mice (three records, 15.8%), then Sprague–Dawley rats (two records, 9.1%), and zebrafish embryos (two records, 9.1%), and lastly, Institute of Cancer Research (ICR) mice (one record, 4.5%). Meanwhile, for cytotoxicity assays, in vitro models (each mentioned once) include the human carcinoma cell lines HEp-2, Caco-2, and T84; mouse melanoma B16F10 cells and human dermal CCD-986sk fibroblasts; L929 fibroblasts; and the HeLa cell line. The most common treatment was the acute toxicity test (eight records, 36.4%), followed by the acute oral toxicity test (two records, 9.1%), the subacute toxicity test (two records, 9.1%), and the MTT assay (two records, 9.1%). Moreover, among the reported species, most of them were nontoxic. Of the rest, five (22.7%) were toxic, one (4.5%) was teratogenic, and one (4.5%) was both toxic (embryotoxic) and teratogenic (Fig. 7).

The data on toxicity and teratogenicity of the three most common species are presented in Table 3. L929 fibroblasts were used as an in vitro model in the study of *I. batatas* (L.) Lam. In contrast, only in vivo models, such as Sprague–Dawley rats, Wistar albino rats, and zebrafish embryos, were used to study *M. charantia* L. and *M. oleifera* Lam. In the MTT assay conducted on L929 fibroblasts by Moura et al. (2020) [26], the extract from the purple variety of *I. batatas* (L.) Lam. did not have cytotoxic potential, while the extract from the white variety did, at a concentration of 1000 µg/mL after 27-h treatment. *M. charantia* L.*,* on the other hand, was considered teratogenic after providing water extracts of the whole unripe fruit to mice [27]. Specifically, *M. charantia* L. affected the development of the reproductive organs, and weight changes were observed in several organs, such as the brain, liver, kidney, lung, spleen, and heart [27]. Meanwhile, *M. oleifera* Lam. was considered nontoxic after an acute oral toxicity test done in Wistar albino rats upon oral administration of an aqueous methanolic leaf extract at a dose of 2000 mg/kg [28]. However, in a study involving Sprague–Dawley rats, given 1000 mg/kg and 3000 mg/kg doses, *M. oleifera* Lam. was considered genotoxic, but when ingested < 1000 mg/kg, it was considered safe [29]. Meanwhile, dilutions of *M. oleifera* Lam. leaf and bark extracts in embryo water resulted in toxic effects on zebrafish embryos [30]. The qualitative synthesis of the studies with data on plants used for anemia in the Philippines is presented in Additional file 1: Table S4.

**Table 3:** Toxicologic and teratogenic data of the most utilized plants used for anemia in the Philippines

Plant species	Study design	Type of study	Treatment	Duration of treatment	Toxicologic and teratogenic effects data	Reference (First Author and Year)
*Ipomoea batatas* (L.) Lam	Preclinical study	In vitro: L929 fibroblasts	MTT assay: aqueous extracts from the leaves of purple and white varieties of *I. batatas* at 50, 100, and 1000 g/mL concentrations were exposed to the cells	27 h	At a concentration of 1000 µg/mL, the extract from the purple variety had no cytotoxic potential, but the extract from the white variety did. The viability of the white variety was 86.37% at 50 µg/mL, 89.58% at 100 µg/mL, and 56.66% at 1000 µg/mL. As a result, the highest concentration proved to be potentially cytotoxic, as it was less than 70%	Moura (2020) [26]
*Momordica charantia* L.	Preclinical study	In vivo: pregnant Sprague–Dawley rats	Water extracts of the whole unripe fruit (unspecified dosage) were administered to eight groups of rats at days 7, 8, 9, 10, 11, 12, 13, and 14 of gestation, respectively	22 days	The extract's teratogenic effects were determined by the day of gestation on which it was administered. Most malformations occurred on days 7, 11, and 13 of gestation. Reproductive organs were affected the most. Weight loss was observed in the brain, liver, kidney, lung, and spleen, while the weight of the heart increased	Uche-Nwachi (2009) [27]
*Moringa oleifera* Lam	Preclinical study	In vivo: Sprague–Dawley rats	Two groups of rats were orally given doses of 1000 mg/kg body weight and 3000 mg/kg body weight, respectively, of aqueous leaf extract. After 48 h, the mice were euthanized, and the femur bone marrow aspirate was examined	14 days	No mortality, behavioral changes, or adverse hematological effects were recorded. It was cytotoxic at 20 mg/mL and genotoxic at supra-supplementation levels of 3000 mg/kg b.wt	Asare (2012) [29]
	Preclinical study	In vivo: Wistar albino rats	Acute oral toxicity test: aqueous-methanolic leaf extract was given orally at a 2000 mg/kg dose for 48 h to establish the median fatal dose	–	No toxic manifestations or mortality at 2000 mg/kg. However, it has potentially toxic effects at higher doses	Okumu (2016) [28]
	Preclinical study	In vivo: zebrafish embryo	10 ml of the different concentrations of leaves and bark extracts (300 ppm, 1500 ppm, 3000 ppm, and 6000 ppm) were prepared by dilution in embryo water	12, 24, and 48 h	Teratogenicity is evident due to the embryos’ low hatchability percentage, lack of or low heartbeat rate, growth retardation, and morphological abnormalities, including yolk deformity and a stunted tail. After 12 and 24 h, it is highly toxic to embryos	David (2016) [30]

## Discussion

The initial literature search provided 402 results and only 20 studies were included in the qualitative analysis after eligibility screening. And as seen in the annual trend of the number of ethnobotanical studies done on plants used for anemia, there remains a big gap to fill in on this data. Since its inception in 1988, few anemia-related ethnobotanical studies have been conducted in the Philippines. Meanwhile, we have obtained the most ethnobotanical studies locally documenting the tribes in Region X (Northern Mindanao) and the Cordillera Administrative Region (CAR). With a rich culture, there are more tribes to explore yet to fill the research gap. These ethnobotanical surveys cover only some of the regions in the Philippines; hence, there is a huge possibility that other plants used for anemia still need to be documented.

Several studies documented the pharmacological effects of bioactive compounds in traditionally used plants in iron deficiency anemia. The anti-anemic activity of the studied ethnomedicinal plants, particularly *I. batatas* (L.) Lam. was evaluated through hematological indices, namely hemoglobin, hematocrit, red blood cell (RBC), mean corpuscular hemoglobin concentration (MCHC), mean corpuscular volume (MCV), and mean corpuscular hemoglobin (MCH), in an experimental study performed on albino rats, where it was observed that the n-hexane leaf extract of *I. batatas* (L.) Lam. triggered regeneration of RBC, hematocrit, hemoglobin, MCV, and MCH [31]. Moreover, in an Indonesian clinical study involving pregnant women, *I. batatas* (L.) Lam. leaf decoction increased the hemoglobin levels [32, 33].

Another study reported how the iron content in the *Moringa* leaves acts as the primary nutrient in hematopoiesis in the spinal cord. Likewise, the protein and amino acid content in *Moringa* leaves acts as hematopoietic growth factors. Its leaves reported a high protein and amino acid content, vital in managing blood cell differentiation and proliferation. Furthermore, the vitamin C content in *Moringa* leaf extract also increases iron absorption in the body [34]. In a study done in male Wistar rats, it was reported that dietary iron from *Moringa* leaves was more effective in overcoming iron deficiency in rats than ferric citrate, which suggests its possible effects on the expression of liver hepcidin mRNA expression [35]. Other medicinal plant families, such as Convovulaceae, Cucurbitaceae, and Amaranthaceae, are believed to contain significant amounts of iron, vitamins, and minerals, which help elevate hemoglobin percentage in the blood.

Literature on the toxicologic and teratogenic data for the three most commonly cited plant species was available. However, the safety and efficacy of these documented medicinal plants in humans still need to be investigated due to the scarcity of preclinical and clinical studies. This systematic review determined the three most cited plant species, which include *I. batatas* (L.) Lam., *M. charantia* L., and *M. oleifera* Lam. In an in vitro study documenting the cytotoxicity of two varieties of *I. batatas* (L.) Lam., the MTT assay showed the cytotoxic potential of the white variant at 1000 μg/ml [26]. Given this result, we suggest further investigating the cytotoxicity of the two variants since the study used lyophilized plants while *I. batatas* (L.) Lam. is commonly cooked. Hence, heat may induce changes in the biochemical properties of the plant [26].

Meanwhile, one study documented the teratogenicity of *M. charantia* L. upon the provision of water extracts of the whole unripe fruit to mice, but the doses of the whole unripe fruit were not specified. Hence, its teratogenicity data was insufficient. Moreover, the study recorded the weights of the internal organs and revealed a reduction in the weight of the brain, liver, lungs, kidneys, and spleen while the heart’s weight increased. It was later concluded that the teratogenicity of the unripe fruit depends on the gestation period during which it was ingested and showed that most malformations occurred on days seven, 11, and 13; however, the duration of treatment should ideally be increased to cover the span of organogenesis. It is also imperative not to draw generalized conclusions based on one study. Therefore, we recommend confirming these data further to validate the results.

Lastly, the toxic dose threshold for *M. oleifera* Lam. should be clarified since one study documented its safety when < 1000 mg/kg per body weight was ingested [29], while another reported that it is nontoxic at a dose of 2000 mg/kg [28]. Hence, its safe dose and toxic dose should be elucidated further. Using a different animal model (i.e., zebrafish embryo), dilutions of *M. oleifera* Lam. leaf and bark extracts were considered toxic. With these, the toxicity and safety of *M. oleifera* Lam. should be further explored. If possible, more varied dose formulations and animal models or cell lines should be examined to ensure its toxicity. The continued use of these plants may pose risks to local tribes, as some have been described as teratogenic and toxic at specific doses. Fortunately, most were deemed safe among the identified species with toxicologic and teratogenic data. However, considering the most used plants, there are dose limits to which the plant can be regarded as safe (e.g., *M. oleifera* Lam.). Hence, precaution should be practiced when using it. Moreover, it is crucial to consider the type of solvents used in plant extraction, as these may be teratogenic and toxic. In the presence of varieties or local variants, genomic studies should also be conducted to ensure the identity of the plant described. When designing toxicologic and teratogenic studies, the method of preparation by which the plant is ingested should be considered. These safety and/or toxicity data (i.e., therapeutic dose), when studied further, should be relayed to the local tribes actively engaged in the use of these plants to lessen the risks of plant toxicity.

With emerging diseases and the demand for low-cost medicines in the current situation, the use of ethnomedicinal plants in the Philippines is inevitable. Even though this systematic review included only articles written in English or Filipino and excluded systematic reviews, literature reviews, letters to the editor, comments, and case reports, we identified the commonly utilized ethnomedicinal plant species, parts of the plants involved, their suggested method of preparation, and the indigenous communities that mainly used these for treating anemia. We also discovered that only a few anemia-related ethnobotanical studies were conducted in the Philippines. Literature is available on the toxicologic and teratogenic data for the three most commonly used plant species. However, their safety and efficacy remain questionable due to preclinical and clinical data scarcity. In addition, the data gathered highlighted the crucial role of various ethnomedicinal plants and how these remain a significant source of medicine among rural dwellers because of the limited means of modern medicine. Notably, most recorded plants grew in the wild and have received little attention in the literature [36]. This means there are still several other unexamined ethnomedicinal plants that can be potentially used for treating anemia. Indeed, there needs to be documentation of the medicinal plants claimed by indigenous communities, which can pave the way for researchers to discover and develop these plant-based medicines [37, 38] in addition to the government’s food fortification efforts in addressing the anemia burden in the country [38]. This review will also help conserve the geographical areas where these plant species naturally thrive and preserve the knowledge and practices related to anemia management. To fill in the gap in the toxicologic and/or teratogenic data, we recommend that the previously documented plants be analyzed first. In turn, this study can guide future research activities to examine active compounds and other potential plant properties that can later be added to the list of medicinal plants officially recognized by the Department of Health—PITAHC and the subsequent incorporation of these into our current health system as complementary medicine.

## Conclusion

This systematic review gathered 20 ethnobotanical studies in the Philippines that reported medicinal plants used to treat anemia. There was an observed increase in ethnobotanical studies through the years. However, a significant research gap exists on the medicinal plants used to treat anemia. There are still regions in the Philippines without ethnobotanical studies related to anemia. This systematic review showed the abundance of medicinal plants used in treating anemia in the Philippines. However, pharmacological and toxicological studies are still needed to determine and verify the safety and efficacy of these medicinal plants in treating anemia in the community. Lastly, this study will guide future researchers to look closely at untapped medicinal plants and review their properties, emphasizing safety and toxicity for humans.

## Supplementary Information

**Additional file 1: Figure S1.** Most common plant parts used (mixed) (A), routes of administration with two separate steps (B), and other modes of preparation (C). **Table S1.** Summary of full-text analysis**.** **Table S2.** Quality assessment of the studies with data on plants used for anemia in the Philippines. **Table S3.** Toxicologic and teratogenic data of plants used for anemia in the Philippines. **Table S4.** Qualitative synthesis of the studies with data on plants used for anemia in the Philippines.

## Data Availability

All data related to this study are included in the manuscript and the additional file.

## Abbreviations

BARMM: Bangsamoro Autonomous Region in Muslim Mindanao
CAR: Cordillera Administrative Region
CALABARZON: Cavite, Laguna, Batangas, Rizal, and Quezon
DOH: Department of Health
ICR mice: Institute of Cancer Research mice
IDA: Iron deficiency anemia
MCH: Mean corpuscular hemoglobin
MCHC: Mean corpuscular hemoglobin concentration
MCV: Mean corpuscular volume
MIMAROPA: Mindoro Occidental, Mindoro Oriental, Marinduque, Romblon, and Palawan
NCR: National Capital Region
PRISMA: Preferred Reporting Items for Systematic Reviews and Meta-analyses
PITAHC: Philippine Institute of Traditional and Alternative Health Care
RBC: Red blood cell
SOCCKSARGEN: South Cotabato, Cotabato, Sultan Kudarat, Sarangani, and General Santos City
