# Supplementary material for: Ethnomedicinal plants used for the prevention and treatment of anemia in the Philippines: a systematic review
Source: Trop Med Health. 2023 May 12;51:27. doi: 10.1186/s41182-023-00515-x (PMC10176884; doi:10.1186/s41182-023-00515-x)
Supplement: Supplementary file 1 — Additional file 1: Figure S1. Most common plant parts used (mixed) (A), routes of administration with two separate steps (B), and other modes of preparation (C). Table S1. Summary of full-text analysis. Table S2. Quality assessment of the studies with data on plants used for anemia in the Philippines. Table S3. Toxicologic and teratogenic data of plants used for anemia in the Philippines. Table S4. Qualitative synthesis of the studies with data on plants used for anemia in the Philippines. [file 41182_2023_515_MOESM1_ESM.docx]

# Supplemental Figure


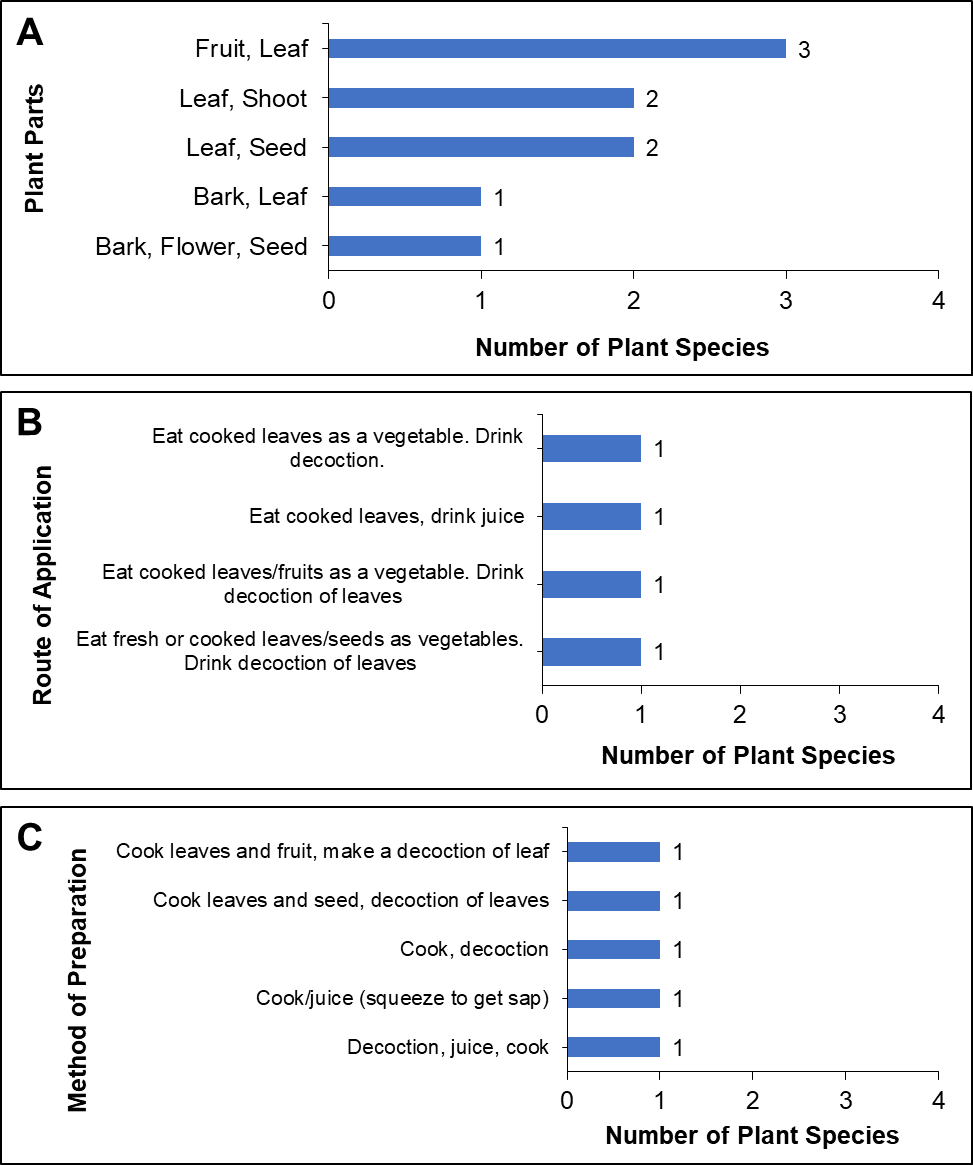


## Supplemental Figure 1. Most common plant parts used (mixed) (A), routes of administration with two separate steps (B), and other modes of preparation (C).

# Supplemental Tables

## Supplemental Table 1. Summary of full-text analysis

| **Species** | **First Author and Year** | **English name** | **Family Name** | **Plant Part Used** | **Method of Preparation** | **Route of Administration** | **Ethnic Group/User** | **Place of Origin** |
| --- | --- | --- | --- | --- | --- | --- | --- | --- |
| *Amaranthus spinosus* L. | Dapar, M.L.G. (2020) [1] | Callaloo, Needle burr, Pigweed, Prickly amaranth, Prickly calalu, Spiny amaranth, Thorny amaranth, Thorny pigweed | Amaranthaceae | Le | De | Dr | Manobos | Agusan del Sur |
| *Antidesma bunius* (L.) Spreng. | Caunca, E.S. (2021) [2] | Buni-berry, Currant tree, Chinese laurel, Maoberry, Queensland cherry, Salamander tree, Wild cherry | Phyllanthaceae | Fr, Le | De | Dr | Local herbalists | Cavite |
| *Caesalpinia sappan* L. | Odchimar, N.M. (2017) [3] | Sappan wood, Brazil wood, Bukkum wood, False sandalwood, Indian redwood, Sappan lignum | Fabaceae | Ro | De | Dr | Talaandig tribe | Bukidnon |
| *Centella asiatica* (L.) Urb. | Rubio, M.M. (2018) [4] | Asiatic pennywort, Indian Hydrocotyle, Pennyworth, Spade leaf, Tiger grass | Apiaceae | Le | De | Ea | Traditional practitioners | North Cotabato |
| *Chamaecostus cuspidatus* (Nees & Mart.) C.D.Specht & D.W.Stev. | de Guzman, A.A. (2020) [5] | Insulin plant | Costaceae | Le, Sh | Wa | Ea | Local herbalists | Zamboanga Sibugay |
| *Cinnamomum mercadoi* Vidal | Naive, M.A.K. (2021) [6] | Kalingag tree | Lauraceae | Ba | De | Dr | Talaandig tribe | Bukidnon |
| *Coleus scutellarioides* (L.) Benth. (Mayana kanapkap variety) | Dapar, M.L.G. (2020) [1] | Butterfly coleus, Coleus, Common coleus, Flame nettle, Joseph's coat, Painted nettle | Lamiaceae | Le | De | Dr | Manobos | Agusan del Sur |
| *Coleus scutellarioides* (L.) Benth. (Mayana pula variety) | Dapar, M.L.G. (2020) [1] | Butterfly coleus, Coleus, Common coleus, Flame nettle, Joseph's coat, Painted nettle | Lamiaceae | Le | De | Dr | Manobos | Agusan del Sur |
| *Cordia dichotoma* G. Forst. | Naive, M.A.K. (2021) [6] | Fragrant manjack, Glue berry, Indian cherry, Lasora, Bird lime tree, Fragrant manjack, Indian cherry, Sebesten plum, Soapberry | Cordiaceae | Ba, Le | De | Dr | Talaandig tribe | Bukidnon |
| *Dendrocnide meyeniana* (Walp.) Chew | Naive, M.A.K. (2021) [6] | - | Urticaceae | Ro | De | Dr | Talaandig tribe | Bukidnon |
| *Euphorbia hirta* L. | Ducusin, M.B. (2017) [7] | Asthma plant, Australian asthma weed, Cat's hair, Hairy spurge, Pill-bearing spurge, Snakeweed | Euphorbiaceae | Wp | De | Dr | Indigenous people | La Union |
| *Ficus botryocarpa* Miq. | Langenberger, G. (2009) [8] | - | Moraceae | Le | De | Dr | Male farmers | Leyte |
| *Flacourtia rukam* Zoll. & Moritzi | Olowa, L.F. (2012) [9] | - | Salicaceae | Ro | De | Dr | Higaonons | Lanao del Norte |
| *Ipomoea aquatica* Forssk. | Olowa, L. (2015) [10] | Potato vine, Swamp cabbage, Swamp morning glory, Water spinach | Convolvulaceae | Le | Co/Ex | Ea/Dr | Maranaos | Lanao del Norte |
| *Ipomoea batatas* (L.) Lam. | Balangcod, T.D. (2018) [11] | Sweetpotato | Convolvulaceae | Sh | Co | Ea | Locals | Benguet |
|  | Olowa, L. (2015) [10] |  |  | Le |  |  | Maranaos | Lanao del Norte |
|  | Ong, H.G. (2014) [12] |  |  |  |  |  | Ati Negritos | Guimaras |
|  | Abe, R. (2013) [13] |  |  |  | Ha |  | Ivatans | Batanes |
|  | Ducusin, M.B. (2017) [7] |  |  |  |  |  | Indigenous people | La Union |
|  | Naive, M.A.K. (2021) [6] |  |  | Le, Sh | De | Dr | Talaandig tribe | Bukidnon |
|  | Alduhisa, G.U. (2019) [14] |  |  | Le | St | Ea, Dr | Subanens | Misamis Occidental |
| *Ipomoea sp.* | Tantengco, O.A.G. (2018) [15] | - | Convolvulaceae | Sh | De | Dr | Aytas | Bataan |
| *Kalanchoe pinnata* (Lam.) Pers. | Dapar, M.L.G. (2020) [1] | Air plant, Cathedral bells, Leaf-of-life, Life plant, Miracle leaf, Miracle plant, Mother of thousands, Live-leaf-of-resurrection plant | Crassulaceae | Le | De | Dr | Manobos | Agusan del Sur |
| *Knema glomerata* (Blanco) Merr. | Paraguison, L. (2020) [16] | - | Myristicaceae | Ba | De | Dr | Manobos | Agusan del Sur |
| *Momordica charantia* L. | Balangcod, T.D. (2015) [17] | African cucumber, Balsam apple, Balsam pear, Bitter cucumber, Bitter gourd, Bitter melon, Tuberculated momordica | Cucurbitaceae | Fr, Le | Co | Ea | Ibalois | Benguet |
|  | Ong, H.G. (2014) [12] |  |  | Fr |  |  | Ati Negritos | Guimaras |
|  | Abe, R. (2013) [13] |  |  | Fr, Le | Co, De | Ea, Dr | Ivatans | Batanes |
|  | Langenberger, G. (2009) [8] |  |  | Le | Ex | Dr | Male farmers | Leyte |
|  | Ducusin, M.B. (2017) [7] |  |  |  | Ha | Ea | Indigenous people | La Union |
|  | Olowa, L. (2015) [10] |  |  | Fr | Ex | Dr | Maranaos | Lanao del Norte |
| *Moringa oleifera* Lam*.* | Balangcod, T.D. (2018) [11] | Ben oil tree, Ben tree, Behn tree, Behen tree, Drumstick tree, Horse-radish tree, Miracle tree | Moringaceae | Le | Co | Ea | Locals | Benguet |
|  | Abe, R. (2013) [13] |  |  | Le, Se | Co, De | Ea, Dr | Ivatans | Batanes |
|  | Ducusin, M.B. (2017) [7] |  |  |  | Co | Ea | Indigenous people | La Union |
|  | Bodner, C.C. (1988) [18] |  |  | Ba, Fl, Se | De | Dr | Bontoc tribe | Mountain Province |
|  | Balangcod, T.D. (2015) [17] |  |  | Le | De, Ex, Co | Ea, Dr | Ibalois | Benguet |
| *Passiflora foetida* L. | Balangcod, T.D. (2011) [19] | - | Passifloraceae | Sh (young) | Co | Ea | Kalanguya tribe | Ifugao |
| *Solanum americanum* Mill. | Naive, M.A.K. (2021) [6] | Black nightshade, American black nightshade, Deadly nightshade, Garden or common nightshade, Poison berry, Small-fruited black nightshade, Wonderberry | Solanaceae | Le | De | Dr | Talaandig tribe | Bukidnon |
| *Solanum nigrum* L. | Abe, R. (2013) [13] | Black nightshade, Deadly nightshade, Garden or common nightshade, Poison berry, Small-fruited black nightshade, Wonderberry | Solanaceae | Le | Co, De | Ea, Dr | Ivatans | Batanes |
| *Terminalia catappa* L. | Abe, R. (2013) [13] | Indian almond, Tropical almond, Umbrella tree | Combretaceae | Fr | Co | Ea | Ivatans | Batanes |
| *Tetragonia tetragonioides* (Pall.) Kuntze | Abe, R. (2013) [13] | Spinach, New Zealand spinach | Aizoaceae | Le | Co | Ea | Ivatans | Batanes |
| *Tinospora cordifolia* (Willd.) Miers | Flores, R.L. (2016) [20] | - | Menispermaceae | Tw | De | Dr | Buyers and sellers | Quiapo |

**Legend**:

**Plant Part Used**: Bark= Ba; Fruit= Fr; Flower= Fl; Leaf= Le; Root= Ro; Seed= Se; Shoot= Sh; Twig= Tw; Whole plant= Wp

**Method of Preparation**: Cook= Co; Decoction= De; Extract= Ex; Harvest= Ha; Steam= St; Wash= Wa

**Route of Administration**: Drink= Dr; Eat= Ea

## Supplemental Table 2. Quality assessment of the studies with data on plants used for anemia in the Philippines.

| **QUALITY ASSESSMENT**  Scoring: Fully compliant= 2 points, Partially compliant= 1 point, Not compliant= 0; N/A= not applicable  Total score: 17–20 = High quality, 11–16= Regular quality, 0–10= Low quality  Questions:  Are the questions or objectives sufficiently described? (Q1)  Is the study design appropriate to answer the study question/s? (Q2)  Is the study area and population sufficiently described? (Q3)  Are the methods described in sufficient detail? (Q4)  Can the study be easily replicated? (Q5)  Is the sample size of informants sufficient or justified? (Q6)  Are the medicinal plants verified by a taxonomist? (Q7)  Did the paper provide appropriate descriptive and quantitative analysis? (Q8)  Are the results reported in sufficient detail? (Q9)  Do the results support the conclusion? (Q10) | | | | | | | | | | | | | | |
| --- | --- | --- | --- | --- | --- | --- | --- | --- | --- | --- | --- | --- | --- | --- |
| **First Author and Year** | **Study** | **Journal** | **Q1** | **Q2** | **Q3** | **Q4** | **Q5** | **Q6** | **Q7** | **Q8** | **Q9** | **Q10** | **Total** | **Quality** |
| Abe, R. (2013) [13] | An ethnobotanical study of medicinal plants and traditional therapies on Batan Island, the Philippines | Journal of Ethnopharmacology | 2 | 2 | 2 | 2 | 2 | 2 | 0 | 2 | 2 | 2 | 18 | High |
| Alduhisa, G.U. (2019) [14] | Ethnomedicinal plants used by the Subanen tribe in two villages in Ozamis City, Mindanao, Philippines. | Pharmacophore | 1 | 2 | 2 | 1 | 2 | 2 | 0 | 2 | 2 | 2 | 16 | Regular |
| Balangcod, T.D. (2011) [19] | Ethnomedical knowledge of plants and healthcare practices among the Kalanguya tribe in Tinoc, Ifugao, Luzon, Philippines. | Indian Journal of Traditional Knowledge | 2 | 2 | 2 | 2 | 2 | 2 | 0 | 2 | 2 | 2 | 18 | High |
| Balangcod, T.D. (2015) [17] | Ethnomedicinal plants in Bayabas, Sablan, Benguet Province, Luzon, Philippines | Electronic Journal of Biology | 2 | 2 | 2 | 2 | 2 | 1 | 0 | 2 | 2 | 1 | 16 | Regular |
| Balangcod, T.D. (2018) [11] | Plants and culture: Plant utilization among the local communities in Kabayan, Benguet Province, Philippines | Indian Journal of Traditional Knowledge | 2 | 2 | 2 | 2 | 2 | 2 | 2 | 2 | 2 | 2 | 20 | High |
| Bodner, C.C. (1988) [18] | A contribution to Bontoc ethnobotany | Economic Botany | 2 | 2 | 2 | 1 | 1 | 0 | 0 | 2 | 1 | N/A | 11 | Regular |
| Caunca, E.S. (2021) [2] | The practice of using medicinal plants by local herbalists in Cavite, Philippines | Indian Journal of Traditional Knowledge | 2 | 2 | 2 | 2 | 2 | 2 | 2 | 2 | 2 | 2 | 20 | High |
| Dapar, M.L.G. (2020) [1] | Quantitative ethnopharmacological documentation and molecular confirmation of medicinal plants used by the Manobo tribe of Agusan del Sur, Philippines | Journal of Ethnobiology and Ethnomedicine | 2 | 2 | 1 | 2 | 2 | 1 | 2 | 2 | 2 | 2 | 18 | High |
| de Guzman, A.A. (2020) [5] | Ethnobotany and physiological review on folkloric medicinal plants of the Visayans in Ipil and Siay, Zamboanga Sibugay, Philippines | International Journal of Herbal Medicine | 1 | 2 | 1 | 1 | 2 | 1 | 0 | 2 | 2 | 2 | 14 | Regular |
| Ducusin, M.B. (2017) [7] | Ethnomedicinal knowledge of plants among the indigenous peoples of Santol, La Union, Philippines | Electronic Journal of Biology | 2 | 2 | 2 | 2 | 2 | 1 | 0 | 2 | 2 | 2 | 17 | High |
| Flores, R.L. (2016) [20] | Ethnomedicinal study of plants sold in Quiapo, Manila, Philippines | Scholars Academic Journal of Biosciences | 2 | 2 | 1 | 1 | 2 | 2 | 0 | 2 | 2 | 2 | 16 | Regular |
| Langenberger, G. (2009) [8] | Ethnobotanical knowledge of Philippine lowland farmers and its application in agroforestry. | Agroforestry Systems | 2 | 2 | 2 | 2 | 2 | 1 | 2 | 2 | 2 | N/A | 17 | High |
| Naive, M.A.K. (2021) [6] | Plants with benefits: Ethnomedicinal plants used by the Talaandig tribe in Portulin, Pangantucan, Bukidnon, Philippines | Indian Journal of Traditional Knowledge | 2 | 2 | 2 | 2 | 2 | 2 | 2 | 2 | 2 | 2 | 20 | High |
| Odchimar, N.M. (2017) [3] | Ethnobotany of medicinal plants used by the Talaandig Tribe in Brgy. Lilingayon, Valencia City, Bukidnon, Philippines. | Asian Journal of Biological and Life Sciences | 2 | 2 | 1 | 1 | 1 | 0 | 0 | 2 | 2 | 2 | 13 | Regular |
| Olowa L. (2015) [10] | Ethnobotanical uses of medicinal plants among the Muslim Maranaos in Iligan City, Mindanao, Philippines | American-Eurasian Network for Scientific Information Journals | 1 | 2 | 1 | 1 | 2 | 1 | 2 | 1 | 2 | 1 | 14 | Regular |
| Olowa, L.F. (2012) [9] | Medicinal plants used by the Higaonon tribe of Rogongon, Iligan City, Mindanao, Philippines | Advances in Environmental Biology | 2 | 2 | 2 | 2 | 2 | 2 | 0 | 2 | 2 | 2 | 17 | High |
| Ong, H.G. (2014) [12] | Quantitative ethnobotanical study of the medicinal plants used by the Ati Negrito indigenous group in Guimaras island, Philippines | Journal of Ethnopharmacology | 2 | 2 | 2 | 2 | 2 | 2 | 2 | 2 | 2 | 2 | 20 | High |
| Paraguison, L. (2020) [16] | Medicinal plants used by the Manobo Tribe of Prosperidad, Agusan Del Sur, Philippines - an Ethnobotanical Survey | Asian Journal of Biological and Life Sciences | 2 | 2 | 2 | 2 | 2 | 2 | 2 | 2 | 2 | 2 | 20 | High |
| Rubio, M.M. (2018) [4] | Ethnomedicinal plants used by traditional healers in North Cotabato, Mindanao, Philippines | Journal of Biodiversity and Environmental Sciences | 2 | 2 | 2 | 2 | 2 | 2 | 0 | 2 | 2 | 2 | 18 | High |
| Tantengco, O.A.G. (2018) [15] | Ethnobotanical survey of medicinal plants used by Ayta communities in Dinalupihan, Bataan, Philippines | Pharmacognosy Journal | 2 | 2 | 2 | 1 | 1 | 1 | 2 | 2 | 2 | 2 | 17 | High |

## Supplemental Table 3. Toxicologic and teratogenic data of plants used for anemia in the Philippines.

| **Plant Species** | **Study Design** | **Type of Study** | **Treatment** | **Duration of Treatment** | **Toxicologic and Teratogenic Effects Data** | **Reference (First Author and Year)** |
| --- | --- | --- | --- | --- | --- | --- |
| *Amaranthus spinosus* L. | Preclinical study | *In vivo*: male Wistar rats | Acute toxicity test: doses up to 2,000 mg/kg of *A. spinosus* methanol extract were administered to rats orally. | 14 days | No behavioral changes or mortality were observed. | Ashok Kumar, B. S. (2014) [21] |
| *Antidesma bunius* (L.) Spreng. | Preclinical study | *In vivo*: ICR (Institute of Cancer Research) mice | Acute toxicity test: ethanolic extract of *A. bunius* fruit up to a dose of 2,000 mg/kg body weight was administered orally. | 14 days | During the experiment, there was no mortality, behavioral, respiratory, or neurologic changes, or changes in body weight, food, or water consumption. Hematological and biochemical profiles in treated mice were normal. When stained with H&E, the morphology of visceral organs in all treated mice was normal. | Muñoz, M. N. M. (2021) [22] |
| *Caesalpinia sappan* L. | Preclinical study | *In vivo*: male Wistar rats | Acute toxicity test: groups 1, 2, and 3 were given 100, 1000, and 2000 mg/kg body weight of C. sappan aqueous extracts, respectively. Furthermore, 2500, 3500, and 5000 mg/kg body weight doses were administered orally during the second phase. | 28 days | No mortality and significant changes in body weight and organ (kidney, liver, and abdomen) weights were observed. | Athinarayanana, G. (2017)[23] |
| *Centella asiatica* (L.) Urb. | Preclinical study | *In vivo*: male and female Swiss mice | Acute toxicity test: acetone extracts from leaves at 100, 500, 1,000, 2,000, and 4,000 mg/kg were administered to the mice. | 24 hours | Water and food consumption were not reduced, and none of the groups died. | Chauhan, P. K. (2012) [24] |
|  |  |  | Subacute toxicity test: acetone extracts from leaves at 100, 500, 1,000, 2,000, and 4,000 mg/kg were administered to the mice daily. | 15 days | There was also a slight change in liver weight, but it did not affect the hepatic enzymes, suggesting that liver function was not altered. |  |
| *Chamaecostus cuspidatus* (Nees & Mart.) C.D.Specht & D.W.Stev. | No toxicologic or teratogenic data | | | | | |
| *Cinnamomum mercadoi* Vidal | No toxicologic or teratogenic data | | | | | |
| *Coleus scutellarioides* (L.) Benth. (Mayana kanapkap variety) | No toxicologic or teratogenic data | | | | | |
| *Coleus scutellarioides* (L.) Benth. (Mayana pula variety) | No toxicologic or teratogenic data | | | | | |
| *Cordia dichotoma* G. Forst. | Preclinical study | *In vivo*: adult female Wistar albino rats | Acute toxicity: methanolic extract of *C. dichotoma* bark (MECD) at 10, 100, and 1000 mg/kg; further, doses of 2,000, 3,000, and 5,000 mg/kg | 24 hours; 48 hours | No gross behavioral changes or other symptoms of toxicity or mortality were observed. The body weight and food consumption of treated rats were normal compared to rats under vehicle control. | Hussain, N. (2020) [25] |
| *Dendrocnide meyeniana* (Walp.) Chew | No toxicologic or teratogenic data | | | | | |
| *Euphorbia hirta* L*.* | Preclinical study | *In vivo*: Swiss albino mice | Acute toxicity test: the plant extract (aerial parts as well as the fresh juice of leaves) was administered to groups of mice at different concentrations (2, 4, 6, 8, and 10 g/kg body weight). | 14 days | No significant changes in behavior, food, or water intake were observed. | Pingale, S. S. (2013) [26] |
| *Ficus botryocarpa* Miq. | No toxicologic or teratogenic data | | | | | |
| *Flacourtia rukam* Zoll. & Moritzi | No toxicologic or teratogenic data | | | | | |
| *Ipomoea aquatica* Forssk. | No toxicologic or teratogenic data | | | | | |
| *Ipomoea batatas* (L.) Lam. | Preclinical study | *In vitro*: L929 fibroblasts | MTT assay: aqueous extracts from the leaves of the purple and white variety of *I. batatas* at concentrations of 50, 100, and 1000 g/mL were exposed to the cells. | 27 hours | At a concentration of 1000 µg/mL, the extract from the purple variety had no cytotoxic potential, but the extract from the white variety did. The viability of the white variety was 86.37% at 50 µg/mL, 89.58% at 100 µg/mL, and 56.66% at 1000 µg/mL. As a result, the highest concentration was potentially cytotoxic, as it was less than 70%. | Moura, I. O. (2020) [27] |
| *Ipomoea* sp*.* | Preclinical study | No toxicologic or teratogenic data | | | | |
| *Kalanchoe pinnata* (Lam.) Pers. | Preclinical study | *In vitro*: human carcinoma cell line HEp-2, Caco-2, and T84 | Phytochemical analysis: leaf aqueous extracts were evaluated at 500 and 1000 μg.mL^-1^ and the flavonoids quercetin and rutin at a concentration of 50 μg/mL. | 24 hours | All cell lines were sensitive to the extract at 1000 μg/mL^-1^ except Hep-2. The flavonoids quercetin and rutin showed no cytotoxicity in any tested cell lines. | Barboza, T. J. D. (2016) [28] |
| *Knema glomerata* (Blanco) Merr. | No toxicologic or teratogenic data | | | | | |
| *Momordica charantia* L. | Preclinical study | *In vivo*: pregnant Sprague-Dawley rats | Water extracts of the whole unripe fruit (unspecified doses) were administered to eight groups of rats at days 7, 8, 9, 10, 11, 12, 13, and 14 of gestation, respectively. | 22 days | The extract is teratogenic depending on the stage of gestation at which it was administered, and the reproductive organs of the pups were most affected. There was a significant reduction in the weights of the brain, liver, kidney, lung, and spleen in the pups, while there was a significant increase in the weight of the heart. | Uche-Nwachi, E. O. (2009) [29] |
| *Moringa oleifera* Lam*.* | Preclinical study | *In vivo*: Sprague-Dawley rats | Two groups of rats were orally given doses of 1000 mg/kg body weight and 3000 mg/kg body weight of aqueous leaf extract. After 48 hours, the mice were euthanized, and the femur bone marrow aspirate was studied. | 14 days | No mortality, behavioral changes, or adverse hematological effects were recorded. It was cytotoxic at 20 mg/mL and genotoxic at supra-supplementation levels of 3000 mg/kg b.wt. | Asare, G. A. (2012) [30] |
|  |  | *In vivo*: Wistar albino rats | Acute oral toxicity test: aqueous-methanolic leaf extract was given at a 2000 mg/kg dose orally for 48 hours to establish the median fatal dose. | - | No toxic manifestations or mortality at 2000 mg/kg. However, it had potential toxic effects at higher doses. | Okumu, M. (2016) [31] |
|  |  | *In vivo*: zebrafish embryo | 10 ml of the different concentrations of leaf and bark extracts (300 ppm, 1500 ppm, 3000 ppm, and 6000 ppm) were prepared by dilution in embryo water. | 12, 24, and 48 hours | Teratogenicity was evident due to the embryos' low hatchability percentage, lack of or low heartbeat rate, growth retardation, and morphological abnormalities, including yolk deformity and a stunted tail. After 12 and 24 hours, it was highly toxic to embryos. | David, C. R. S. (2016) [32] |
| *Passiflora foetida* L. | Preclinical study | *In vivo*: Wistar rats | Chronic toxicity test: four groups of rats were administered with ethanol extract (unspecified) at doses of 16, 160, 800, and 1,600 mg/kg respectively, daily. | 6 months | The extract had no effect on the animals' body weights, food intake, or relative organ weights, nor did it cause abnormal changes in hematological or biochemical values. Histopathological alterations in the various organs of all the extract-treated groups were insignificant, except for the adrenal glands of the highest-dose male group, which showed fatty infiltration in the cortex. However, this phenomenon might be a physiological rather than a pathological change. | Chivapat, S. (2011) [33] |
| *Solanum americanum* Mill. | Preclinical study | *In vivo*: male Wistar rats | Acute toxicity test: α-Solamargine isolated from *S. americanum* fruits was injected into the rats at a dose of 10 mg/kg body weight daily. | 5 days | The extract showed dose-dependent toxicity. Within the first two hours of α-solamargine inoculation, it also caused muscle contraction and dizziness, but no bleeding from the eyes or nose was observed. | Al Chami, L. (2003) [34] |
|  |  |  | Subchronic toxicity test: α-Solamargine isolated from *S. americanum* fruits was injected into the rats at doses of 15. 25 and 35 mg/kg body weight | 24 hours | There was no toxic effect on vital organ tissues. |  |
| *Solanum nigrum* L. | Preclinical study | *In vivo*: Swiss mice | Acute toxicity test: 5 mg/kg of ethanolic extract of the fruit was administered to the mice. If no mortality was observed, doses of 100, 300, and 1,500 mg/kg were administered. | 3 days | No mortality was observed. | Kaushik, D. (2009) [35] |
| *Terminalia catappa* L. | Preclinical study | *In vivo*: male albino Wistar rats | Acute oral toxicity test: aqueous leaf extract at 1,000, 2,500, and 5,000 mg/kg body weight was administered once to the rats. | 14 days | Treatment at doses up to 5000 mg/kg b.wt resulted in no clinical toxicity, morbidity, or mortality. | Iheagwam, F. N. (2021) [36] |
|  |  |  | Sub-acute toxicity study: aqueous leaf extract  administered daily by gastric intubation at a dose of 200, 400, and 800 mg/kg body weight | 28 days | Treatment daily changed body weight gain, plasma alkaline phosphatase activity, and albumin concentration significantly (p<0.05). |  |
| *Tetragonia tetragonioides* (Pall.) Kuntze | Preclinical study | *In vitro*: Mouse melanoma B16F10 cells and human dermal CCD-986sk fibroblasts | MTT assay: ferulic acid isolated from *T. tetragonioides* at 5, 10, 20, 25, or 50 μg/mL concentrations were exposed to the cells. | 48 hours | When compared to control cells, cell viability decreased dose-dependent after treatment with 5, 10, 20, 25, or 50 μg/mL of ferulic acid. At 20 μg/mL, B16F10 cells showed 83.0% viability, and CCD-986sk cells showed 85.0% viability. | Park, H. J. (2018) [37] |
| *Tinospora cordifolia* (Willd.) Miers | Preclinical study | *In vivo*: zebrafish embryos | Zebrafish embryo lethality assay: leaves and bark extracts were examined in zebrafish embryos at 5% and 10%. | 48 hours | The extract showed dose-dependent embryotoxic effects. Among the two extracts, 5% and 10% of the leaf extract recorded the highest mortality of 100%, while the bark extract showed a mortality of 11.11% and 33.33% at 5% and 10% concentrations, respectively. However, no mortality was noted in embryos exposed at lower concentrations for up to 48 hours. In addition, teratogenic effects were also observed in embryos: delayed growth, limited movement, a slightly detached tail from the yolk, and underdeveloped eyes. However, spontaneous movement and very active embryos due to the completely detached tail were observed at 0.5% or lower concentrations of both extracts, which were apparently comparable to the control embryos. | Romagosa, C. (2016) [38] |
|  |  | *In vitro*: HeLa cell line | Pratt and Wills test: 0, 1, 2, 4, 5, 6, or 8 mg/ml⁻¹ of *T. cordifolia* stem extracts were exposed to the cells. | 4 hours | The cytotoxic effect of extract increased in a dose-dependent manner in HeLa cells, and an 80% decline in the surviving fraction was observed for the 4 hours of treatment duration. | Jagetia, G. C. (2006) [39] |

## Supplemental Table 4. Qualitative synthesis of the studies with data on plants used for anemia in the Philippines.

| **First Author and Year** | **Study Design** | **Province** | **Informants** | **Sample Size** | **Number of Plant Species Used for Anemia** |
| --- | --- | --- | --- | --- | --- |
| Abe R. (2013) [13] | Observational Study | Batanes | Ivatan | 116 | 6 |
| Alduhisa G.U. (2019) [14] | Observational Study | Misamis Occidental | Subanen | 83 | 1 |
| Balangcod T.D. (2011) [19] | Observational Study | Ifugao | Kalanguya tribe | 150 | 1 |
| Balangcod T.D. (2015) [17] | Observational Study | Benguet | Ibalois | 80 | 2 |
| Balangcod T.D. (2018) [11] | Observational Study | Benguet | Locals | 107 | 2 |
| Bodner C.C. (1988) [18] | Observational Study | Mt. Province | Bontoc tribe | Not stated | 1 |
| Caunca E.S. (2021) [2] | Observational Study | Cavite | Local herbalists | 94 | 1 |
| Dapar M.L.G. (2020) [1] | Observational Study | Agusan del Sur | Manobos | 335 | 4 |
| de Guzman A.A. (2020) [5] | Observational Study | Zamboanga Sibugay | Local herbalists | 30 | 1 |
| Ducusin M.B. (2017) [7] | Observational Study | La Union | Indigenous people | 40 | 4 |
| Flores R.L. (2016) [20] | Observational Study | Quiapo | Buyers and sellers of medicinal plants | 39 | 1 |
| Langenberger G. (2009) [8] | Observational Study | Leyte | Male farmers | 6 | 2 |
| Naive M.A.K. (2021) [6] | Observational Study | Bukidnon | Talaandig tribe | 19 | 5 |
| Odchimar N.M. (2017) [3] | Observational Study | Bukidnon | Talaandig tribe | Not stated | 1 |
| Olowa L.F. (2012) [9] | Observational Study | Lanao del Norte | Higaonons | 65 | 1 |
| Olowa L. (2015) [10] | Observational Study | Lanao del Norte | Maranaos | 228 | 3 |
| Ong H.G. (2014) [12] | Observational Study | Guimaras | Ati Negritos | 65 | 2 |
| Paraguison L. (2020) [16] | Observational Study | Agusan del Sur | Manobos | 144 | 1 |
| Rubio M.M. (2018) [4] | Observational Study | North Cotabato | Traditional practitioners | 20 | 1 |
| Tantengco O.A.G. (2018) [15] | Observational Study | Bataan | Aytas | 26 | 1 |

## References

[1] M.L.G. Dapar, G.J.D. Alejandro, U. Meve, S. Liede-Schumann, Quantitative ethnopharmacological documentation and molecular confirmation of medicinal plants used by the Manobo tribe of Agusan del Sur, Philippines, J. Ethnobiol. Ethnomed. 16 (2020) 1–60. https://doi.org/10.1186/S13002-020-00363-7.

[2] E.S. Caunca, L.O. Balinado, The practice of using medicinal plants by local herbalists in Cavite, Philippines, Indian J. Tradit. Knowl. 20 (2021) 335–343.

[3] N.M.O. Odchimar, O.M. Nuñeza, M.M. Uy, W.T.P.S.K. Senarath, Ethnobotany of Medicinal Plants used by the Talaandig Tribe in Brgy. Lilingayon, Valencia City, Bukidnon, Philippines., Asian J. Biol. Life Sci. 6 (2017) 358–364.

[4] M. Rubio, N. Arcebal, Ethnomedicinal plants used by traditional healers in North Cotabato, Mindanao, Philippines, J. Biodivers. Environ. Sci. 13 (2018) 74–82.

[5] A.A. de Guzman, C.E.V.A. Jamanulla, A.M. Sabturani, G. Madjos, Ethnobotany and physiological review on folkloric medicinal plants of the Visayans in Ipil and Siay, Zamboanga Sibugay, Philippines, Philipp. Int. J. Herb. Med. 8 (2020) 8–16.

[6] M.A.K. Naive, S.D.A. Binag, G.J.D. Alejandro, Plants with benefits: Ethnomedicinal plants used by the Talaandig tribe in Portulin, Pangantucan, Bukidnon, Philippines, Indian J. Tradit. Knowl. 20 (2021) 754–766. https://doi.org/10.56042/ijtk.v20i3.26584.

[7] M.B. Ducusin, Ethnomedicinal Knowledge of Plants among the Indigenous Peoples of Santol, La Union, Philippines, Electron. J. Biol. 13 (2017) 360–382.

[8] G. Langenberger, V. Prigge, K. Martin, B. Belonias, J. Sauerborn, Ethnobotanical knowledge of Philippine lowland farmers and its application in agroforestry, Agrofor. Syst. 76 (2009) 173–194. https://doi.org/10.1007/s10457-008-9189-3.

[9] L. Olowa, M.A. Torres, E. Aranico, C. Demayo, Medicinal plants used by the Higaonon tribe of Rogongon, Iligan City, Mindanao, Philippines, Adv. Environ. Biol. 6 (2012) 1442–1449.

[10] L. Olowa, C.G. Demayo, Ethnobotanical Uses of Medicinal Plants among the Muslim Maranaos in Iligan City, Mindanao, Philippines, Adv. Environ. Biol. 9 (2015) 204–215.

[11] T. Balangcod, K. Balangcod, Plants and culture: Plant utilization among the local communities in Kabayan, Benguet Province, Philippines, Indian J. Tradit. Knowl. 17 (2018) 609–622.

[12] H.G. Ong, Y.-D. Kim, Quantitative ethnobotanical study of the medicinal plants used by the Ati Negrito indigenous group in Guimaras island, Philippines, J. Ethnopharmacol. 157 (2014) 228–242. https://doi.org/https://doi.org/10.1016/j.jep.2014.09.015.

[13] R. Abe, K. Ohtani, An ethnobotanical study of medicinal plants and traditional therapies on Batan Island, the Philippines, J. Ethnopharmacol. 145 (2013) 554–565. https://doi.org/10.1016/J.JEP.2012.11.029.

[14] G.U. Alduhisa, C.G. Demayo, Ethnomedicinal plants used by the Subanen tribe in two villages in Ozamis City, Mindanao, Philippines, Pharmacophore. 10 (2019) 28–42.

[15] O.A.G. Tantengco, M.L.C. Condes, H.H.T. Estadilla, E.M. Ragragio, Ethnobotanical Survey of Medicinal Plants used by Ayta Communities in Dinalupihan, Bataan, Philippines, Pharmacogn. J. 10 (2018) 859–870. https://doi.org/10.5530/pj.2018.5.145.

[16] L. Paraguison, D.N. Tandang, G.J. Alejandro, Medicinal Plants used by the Manobo Tribe of Prosperidad, Agusan Del Sur, Philippines: an Ethnobotanical Survey, Asian J. Biol. Life Sci. 9 (2020) 326–333. https://doi.org/10.5530/ajbls.2020.9.49.

[17] T. Balangcod, K. Balangcod, Ethnomedicinal Plants in Bayabas, Sablan, Benguet Province, Luzon, Philippines, Electron. J. Biol. 11 (2015) 63–73.

[18] C.C. Bodner, R.E. Gereau, A contribution to Bontoc ethnobotany, Econ. Bot. 42 (1988) 307–369. https://doi.org/10.1007/BF02860159.

[19] T. Balangcod, A.K. Balangcod, Ethnomedical knowledge of plants and healthcare practices among the Kalanguya tribe in Tinoc, Ifugao, Luzon, Philippines, Indian J. Tradit. Knowl. 10 (2011) 227–238.

[20] R.L. Flores, R.A. Legario, D.B.M. Malagotnot, K. Peregrin, R.A. Sato, E.R. Secoya, Ethnomedicinal Study of Plants Sold in Quiapo, Manila, Philippines, 206. 4 (2016) 359–365.

[21] B.S. Ashok Kumar, K. Lakshman, C. Velmurugan, S.M. Sridhar, S. Gopisetty, Antidepressant Activity of Methanolic Extract of Amaranthus Spinosus, Basic Clin. Neurosci. 5 (2014) 11–17.

[22] M.N.M. Muñoz, U.G. Alvarado, J.I.L. Reyes, K. Watanabe, Acute oral toxicity assessment of ethanolic extracts of Antidesma bunius (L.) Spreng fruits in mice, Toxicol. Reports. 8 (2021) 1289–1299. https://doi.org/https://doi.org/10.1016/j.toxrep.2021.06.010.

[23] G. Athinarayanana, A.J.A. Ranjitsingh, A. Usha Raja Nanthini, C. Padmalatha, Toxicological studies of Caesalpinia sappan wood derived dye in Wister albino rats, Food Sci. Hum. Wellness. 6 (2017) 34–38. https://doi.org/https://doi.org/10.1016/j.fshw.2016.10.004.

[24] P. Chauhan, V. Singh, Acute and Subacute Toxicity study of the Acetone Leaf extract of Centella asiatica in Experimental Animal Models, Asian Pac. J. Trop. Biomed. 2 (2012) S511–S513. https://doi.org/10.1016/S2221-1691(12)60263-9.

[25] N. Hussain, B. Kakoti, M. Rudrapal, Z. Rahman, M. Rahman, D. Chutia, K. Sarwa, Anti-Inflammatory and Antioxidant Activities of Cordia dichotoma Forst, Biomed. Pharmacol. J. 13 (2020) 2093–2099. https://doi.org/10.13005/bpj/2090.

[26] S.S. Pingale, Evaluation of acute toxicity study for Euphorbia hirta, Int. J. Bioassays. 2 (2013) 329–332.

[27] I.O. Moura, C.C. Santana, Y.R.F. Lourenço, M.F. Souza, A.R.S.T. Silva, S.S. Dolabella, A.M. de Oliveira e Silva, T.B. Oliveira, M.C. Duarte, A.S. Faraoni, Chemical Characterization, Antioxidant Activity and Cytotoxicity of the Unconventional Food Plants: Sweet Potato (Ipomoea batatas (L.) Lam.) Leaf, Major Gomes (Talinum paniculatum (Jacq.) Gaertn.) and Caruru (Amaranthus deflexus L.), Waste and Biomass Valorization. 12 (2020) 2407–2431. https://doi.org/10.1007/s12649-020-01186-z.

[28] J. Barboza, F. Andréa, C. Ana, A. Norma, Cytotoxic, antibacterial and antibiofilm activities of aqueous extracts of leaves and flavonoids occurring in Kalanchoe pinnata (Lam.) Pers., J. Med. Plants Res. 10 (2016) 763–770. https://doi.org/10.5897/JMPR2016.6260.

[29] E.O. Uche-Nwachi, C. McEwen, Teratogenic effect of the water extract of bitter gourd (Momordica charantia) on the Sprague Dawley rats, African J. Tradit. Complement. Altern. Med. AJTCAM. 7 (2009) 24–33. https://doi.org/10.4314/AJTCAM.V7I1.57228.

[30] G.A. Asare, B. Gyan, K. Bugyei, S. Adjei, R. Mahama, P. Addo, L. Otu-Nyarko, E.K. Wiredu, A. Nyarko, Toxicity potentials of the nutraceutical Moringa oleifera at supra-supplementation levels, J. Ethnopharmacol. 139 (2012) 265–272. https://doi.org/10.1016/J.JEP.2011.11.009.

[31] M. Okumu, J. Mbaria, L. Kanja, D. Gakuya, S. Kiama, F. Okumu, P. Okumu, Acute toxicity of the aqueous-methanolic Moringa oleifera (Lam) leaf extract on female Wistar albino rats, Int. J. Basic Clin. Pharmacol. 5 (2016) 1856–1861. https://doi.org/10.18203/2319-2003.ijbcp20163153.

[32] C.R.S. David, A. Angeles, R.C. Angoluan, J.P.E. Santos, E.S. David, R.M.R. Dulay, Moringa oleifera (Malunggay) Water Extracts Exhibit Embryotoxic and Teratogenic Activity in Zebrafish (Danio rerio) Embryo Model, Der Pharm. Lett. 8 (2016). https://doi.org/10.13140/RG.2.2.13657.34403.

[33] S. Chivapat, M. Bunjob, A. Shuaoprom, J. Bansidhi, P. Chavalittumrong, A. Rangsripipat, P. Sincharoenpokai, Chronic toxicity of Passiflora foetida L. extract, Int. J. Appl. Res. Nat. Prod. 4 (2011) 24–31.

[34] L. Al Chami, R. Méndez, B. Chataing, J. O’Callaghan, A. Usubillaga, L. LaCruz, Toxicological effects of alpha-solamargine in experimental animals, Phytother. Res. 17 (2003) 254–258. https://doi.org/10.1002/PTR.1122.

[35] D. Kaushik, V. Jogpal, P. Kaushik, S. Khokra, C. Sharma, K.R. Aneja, Evaluation of activities of Solanum nigrum fruit extract, Arch. Appl. Sci. Res. 1 (2009) 43–50.

[36] F.N. Iheagwam, C.O. Okeke, O.C. De Campos, B.E. Adegboye, O.O. Ogunlana, S.N. Chinedu, Toxicopathological, proinflammatory and stress response evaluation of Terminalia catappa extract in male Wistar rats, Toxicol. Reports. 8 (2021) 1769–1776. https://doi.org/10.1016/J.TOXREP.2021.10.005.

[37] H.J. Park, J.H. Cho, S.H. Hong, D.H. Kim, H.Y. Jung, I.K. Kang, Y.J. Cho, Whitening and anti-wrinkle activities of ferulic acid isolated from Tetragonia tetragonioides in B16F10 melanoma and CCD-986sk fibroblast cells, J. Nat. Med. 72 (2018) 127–135. https://doi.org/10.1007/S11418-017-1120-7.

[38] C. Romagosa, E.S. David, R.R. Dulay, Embryo-toxic and teratogenic effects of Tinospora cordifolia leaves and bark extracts in Zebrafish (Danio rerio) embryos, Asian J. Plant Sci. Res. 6 (2016) 37–41.

[39] G.C. Jagetia, S.K. Rao, Evaluation of Cytotoxic Effects of Dichloromethane Extract of Guduchi (Tinospora cordifolia Miers ex Hook F & THOMS) on Cultured HeLa Cells, Evid. Based. Complement. Alternat. Med. 3 (2006) 267–272. https://doi.org/10.1093/ECAM/NEL011.
